# "It Depends on What They Experience in Each Health Facility. Some Are Satisfied, Others Are Not." A MixedMethods Exploration of Health Workers’ Attitudes Towards Performance-Based Financing in Burkina Faso

**DOI:** 10.34172/ijhpm.2020.61

**Published:** 2020-05-04

**Authors:** Julia Lohmann, Jean-Louis Koulidiati, Serge MA Somda, Manuela De Allegri

**Affiliations:** ^1^London School of Hygiene & Tropical Medicine, London, UK.; ^2^Institute of Global Health, Heidelberg University Hospital and Medical Faculty, Heidelberg, Germany.; ^3^Centre MURAZ, Bobo-Dioulasso, Burkina Faso.; ^4^UFR/ST, Université Nazi Boni, Bobo-Dioulasso, Burkina Faso.

**Keywords:** Performance-Based Financing,, Burkina Faso, Health Workers, Satisfaction, Knowledge

## Abstract

**Background:** Evidence emerging from qualitative studies suggests the existence of substantial variation in how health workers experience performance-based financing (PBF) within the same setting. To date, however, no study has quantified or systematically explored this within-setting heterogeneity. Considering that differences in health workers’ affective reactions to PBF likely constitute an important element mediating the effectiveness of PBF in improving health service delivery, systematic and tangible information will be highly valuable to policy-makers and program managers who aim to maximize positive impacts of PBF. Our study aimed at contributing to filling this gap in knowledge by quantifying health workers’ knowledge of, satisfaction with, and perceptions of PBF in Burkina Faso, and exploring factors associated with heterogeneity therein.

**Methods:** The study employed a post-intervention cross-sectional explanatory mixed methods study design with a dominant quantitative component – a structured survey to a total of 1314 health workers from 396 intervention health facilities – and a small and focused qualitative component – key informant interviews with 5 program managers – to triangulate and further elucidate the quantitative findings. Quantitative data were analyzed descriptively as well as using 3-level mixed-effects models. Qualitative data were analyzed in a largely deductive process along the quantitative variables and results.

**Results:** Health workers were on average moderately satisfied with PBF overall, with a slight tendency towards the positive and large variation between individuals. Two-thirds of health workers did not have adequate basic knowledge of key PBF elements. Perceived fairness of the performance evaluation process, of the bonus distribution process, and satisfaction with the individual financial bonuses varied dramatically between respondents. Factors associated with heterogeneity in knowledge, satisfaction, and fairness perceptions included responsibility at the facility, general work attitudes, management factors, and training in and length of exposure to PBF.

**Conclusion:** Findings imply that investments into staff training on PBF and manager training on organizational change processes might be beneficial to positive staff attitudes towards PBF, which in turn would likely contribute to improving the effectiveness of PBF.

## Background

Performance-based financing (PBF) has received much attention as a strategy to strengthen health service delivery in low- and middle-income countries in recent years. Studies on the impact of PBF on health service utilization and quality have shown very mixed results.^1-3^ Qualitative studies have identified a large variety of factors related to intervention design, implementation process, and implementation contexts facilitating or hindering PBF impact.^1,4^ Given that one of the key mechanisms by which PBF is assumed to effect change is by motivating health workers to perform better at work,^5-7^ some studies have explored health workers’ experiences of and satisfaction with PBF. Key themes identified fairly consistently across countries include positive perceptions on changes in the work environment^8-16^; dissatisfaction with common delays in payment of PBF bonuses^10,11,14,16,18^; and perceived unfairness of performance verification and reward distribution.^10-20^

Qualitative studies further suggest important variation in health workers’ experiences of and satisfaction with PBF within the same country. For instance, in only one out of 3 districts in Sierra Leone did health workers report positive views on being paid according to their performance.^10^ In Malawi, dissatisfaction with the individual financial incentives was more pronounced in district hospitals with large staff numbers than in small health centers with only a few staff members.^13^ In Tanzania, large differences in satisfaction with incentive payments were reported between staff in the reproductive health department, who were the primary target of PBF and received a higher share of the PBF revenue, and other staff.^18^

 To date, however, evidence on health workers’ experiences and satisfaction with PBF stems exclusively from qualitative studies with a small scope. Moreover, no study has systematically explored how health workers’ experiences and satisfaction within the same country and intervention vary to our knowledge. Considering that differences in health workers’ reactions to PBF likely constitute an important element mediating the effectiveness of PBF in improving health service delivery, systematic and tangible information will be highly valuable to policy-makers and program managers who aim to maximize positive impacts of PBF. Our study aimed at contributing to filling this gap in knowledge by quantifying health workers’ knowledge of, satisfaction with, and perceptions of towards PBF in Burkina Faso, and exploring factors associated with variation in knowledge, satisfaction, and perceptions. In the following, we will use the term “heterogeneity” for such variation in knowledge, satisfaction, and perceptions between respondents.

**Figure 1:**
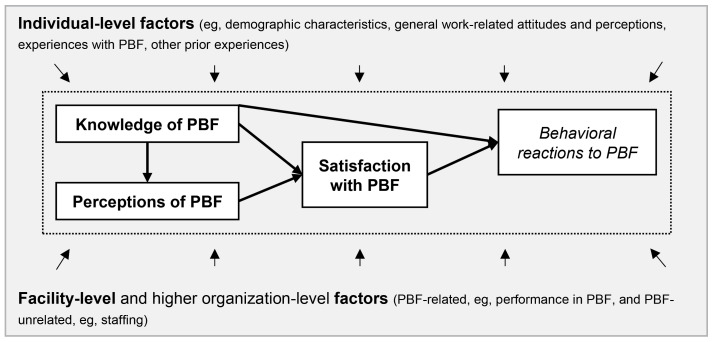
Conceptual Model. Abbreviation: PBF, performance-based financing.

Figure 1 illustrates the understanding of how knowledge, perceptions, and satisfaction shape health workers’ behavioral reaction to PBF which guided our study. This understanding is grounded in the above-reviewed literature. In essence, we assume that the extent to which health workers change their workplace behavior in response to PBF is to a substantial extent determined by health workers’ satisfaction with PBF, in that individuals’ likelihood to change their behavior in alignment with PBF objectives is higher the higher their satisfaction with the intervention, other factors held constant. We further assume that satisfaction, in turn, is influenced by health workers’ levels of knowledge of the intervention and judgements regarding procedural fairness, particularly such in relation to performance evaluation and the individual bonus payment. We assume that the higher knowledge levels and fairness perceptions are, the more satisfied an individual will be. Finally, we assume that knowledge, fairness evaluations, and satisfaction are shaped by a large number of factors at the individual and organizational level, such as general work-related attitudes and the work environment into which PBF is implemented. In line with the mixed-methods and exploratory nature of our work, Figure 1 is meant as an illustration of key factors and relationships aiming at guiding the study, but leaving room for detailed factors and relationships to emerge from the data, rather than as a deterministic model of variables and relationships to be tested.

## Methods

###  Study Setting

Despite improvements over the last years, Burkina Faso continues to suffer from a high burden of morbidity and mortality, with a maternal mortality ratio of 371 per 100000 live births and an under-five mortality rate of 88.6 per 1000 live births (2015).^21^ Health services are provided primarily by the public sector in a multi-tier district health system.^22^ Health facilities upkeep their operations through a mix of government in-kind inputs and revenues from user fees and drug sales.^23^ Formal healthcare service utilization rates have improved substantially in recent years, but remain below target.^24^ Quality of health services, however, is often substandard^25-27^ for reasons including low pay, substandard infrastructure and equipment, poor supervision, shortages in drugs and other supplies, and few incentives for high performance.^22,28-30^

###  Performance-Based Financing in Burkina Faso

Against this background, PBF was first introduced in 2011 as a pilot scheme in 3 health districts to improve access to and quality of care. Given an initially promising evaluation,^31^ PBF was scaled up to another 12 districts between 2014 and 2018, implemented by the Ministry of Health (MoH) with financial support by the World Bank’s Health Results Innovation Trust Fund. The intervention and its background and context are described in detail elsewhere.^32,33^ Although the primary objective was to improve utilization and quality of maternal and child health services, the intervention effectively included a broad range of primary- and secondary-level services, including also curative care, TB, and HIV services. In brief, health facilities signed contracts with the MoH stipulating the services purchased by PBF, a comprehensive list of quality indicators, and payment modalities. Facilities reported volume of provided services on a monthly basis. Reports were then verified by an external agency and facilities subsequently paid a pre-defined amount (‘subsidies’) for each service provided. Subsidies per provided service ranged from 100 FCFA (≈ 0.15 EUR) for curative outpatient consultations to 8500 FCFA (≈ 13 EUR) for a cured tuberculosis case. Facilities were further categorized into 9 equity categories based on staffing levels and remoteness, and less privileged facilities received proportionally higher subsidies. Quality was verified by the District Health Management Teams on a quarterly basis. If quality scores surpassed 50% (later changed to 60%) of the maximum, facilities were paid a quality bonus proportional to their service volume and quality level. PBF payments came on top of pre-existing financing structures. Initially, facilities were free to spend PBF funds as they wished, for facility-related investments or as staff bonuses. From October 2016 on, to encourage more intensive investments, staff bonuses were limited to 60% of the revenue from PBF, whereas at least 40% had to be invested to improve the infrastructure or equipment of the health facility. Facilities were provided with a financial management tool called ‘outil d’indice.’ This also included a calculator to determine bonus amounts for individual staff members, based on 5 criteria. In some health facilities, following a randomization process in the context of an impact evaluation,^32^ the standard PBF was further complemented with measures intended to increase equity in impact.

The impact evaluation of the extended PBF trial showed limited overall effects of PBF, with positive impact only on the utilization of facility-based delivery and postnatal care as well as on certain input dimensions of quality of care, but no impact on the utilization of other services or process quality.^34^ A process evaluation of the first twelve months of implementation underlined that although the intervention was implemented as planned in most respects, there were a number of important challenges, most notably delays in setting up the verification process and in payment of the subsidies.^35,36^

###  Study Design

We used a post-intervention cross-sectional explanatory mixed methods study design with a dominant quantitative component and a small and focused qualitative component. The quantitative component employed a structured survey to health workers in all intervention health facilities to quantify the elements printed in bold in Figure 1, namely health workers’ satisfaction with PBF overall as well as knowledge and perceptions related to the key issues having emerged repeatedly in previous research, performance evaluation and individual bonus payments. The quantitative survey further served to quantify associations with key individual- and facility-level determinants. The qualitative component employed key informant interviews with program managers to triangulate and further elucidate the quantitative findings. It also served to capture factors and dynamics which we had not included in the quantitative survey, allowing us to place quantified associations into context. Qualitative interviews were performed after a descriptive analysis of the quantitative data, and results then used to further inform quantitative analyses of heterogeneity in knowledge, perceptions, and satisfaction. Specifically, results from the qualitative study component led us to obtain and include in the final models additional quantitative data on facility performance as described in more detail below.

###  Quantitative Study Component

####  Design and Sample

Quantitative data were collected in the context of the above-mentioned impact evaluation. The study design and sampling procedures are described in detail in De Allegri et al.^32^ In brief, the study included all 396 primary-level healthcare facilities in all 12 purposely selected intervention health districts that newly received PBF in 2014. In line with the specific objectives set for the study presented in this paper, we only used endline data, collected between April and June 2017, approximately 3 years after the introduction of PBF.

In each health facility, we included all clinical skilled personnel who had worked at the health facility for at least 3 months and who were present on the day of the study team visit, resulting in a total of 1314 health workers (health workers per facility: mean = 3.3, sd = 1.7, min = 1, max = 11). Table 1 provides an overview over the distribution of basic demographic and PBF-related characteristics in the sample.

**Table 1:** Quantitative Sample Characteristics

	**No.**	**%**
Total	1314	100
Gender		
Female	689	52.4
Male	625	47.6
Health worker type*		
Nurse	522	39.7
Midwife	153	11.7
Assistant midwife	330	25.1
AIS	309	23.5
Responsibility		
Health facility in-charge	414	31.5
Staff member	900	68.5
PBF exposure		
From the intervention start	767	58.4
From later	547	41.6
	**Mean**	**SD**
Years in healthcare service	5.9	5.0

Abbreviations: PBF, performance-based financing; AIS, Agent Itinérant de Santé (preventive services and outreach); SD, standard deviation. ^a^ Nurse: Infirmier Diplômé d’Etat, Infirmier breveté; Midwife: Sage-Femme d’Etat/Maïeuticien d’Etat; Assistant midwife: Accoucheuse Brevetée, Accoucheuse Auxilliaire.

####  Data Sources and Data Collection Process

Data was collected with a French-language structured survey administered to all sampled health workers by trained interviewers. The survey assessed overall satisfaction with the PBF intervention as well knowledge and perceptions of the performance evaluation process and the individual incentives as outlined above (6 variables in total, referred to as “outcome variables” in the following). The questionnaire also included questions on demographics, working conditions and perceived working environment, motivation, and clinical knowledge. Questionnaire sections pertaining to satisfaction, attitudes, perceptions, and other psychological aspects were administered in the hybrid mode described in Lohmann et al,^37^ whereby interviewers read questions, statements, and answer options to the respondents, but respondents entered their answers themselves into the tablet computers used for data collection so as to maximize perceived confidentiality and reduce answer biases. We extracted data on facility catchment population, staffing levels, and patient numbers from a facility assessment also conducted within the context of the impact evaluation. To complement the quantitative analysis, we further obtained program data on facility performance on quality indicators and on facility equity categories. Outcome variables as well as potential determinants of heterogeneity are aligned with the conceptual understanding described earlier and detailed in Table 2.

**Table 2:** Variables and Their Measurement

**Variable**	**Measurement**		**Data Source**
	**Question**	**Response **	
** Outcome Variables: PBF Knowledge, Satisfaction, and Perceptions **			
Overall satisfaction with PBF	“How satisfied are you with PBF overall?”	Scale from 0 “not satisfied at all” to 10 “completely satisfied”	Health worker survey
Knowledge performance evaluation	Correct recall of result of last quality verification (+/- 5 points on the 0-100 scale used by the PBF program)	0 = did not know or incorrectly recalled last result; 1 = correctly recalled last result	
Perceived fairness performance evaluation^a^	“Did you find this result fair or unfair considering the performance of your health facility?”	Scale from 0 “not fair at all” to 10 “completely fair”	
Knowledge bonus distribution	Correct recall of who set the bonus distribution mode and according to which criteria bonuses are distributed (min 4 out of 5)	0 = insufficient knowledge; 1 = sufficient knowledge	
Perceived fairness bonus distribution	“Do you think that the system of bonus distribution among staff members is fair or unfair?”	Scale from 0 “not fair at all” to 10 “completely fair”	
Satisfaction with earnings from PBF^b^	“How satisfied are you with the bonus payments you receive?”	Scale from 0 “not satisfied at all” to 10 “completely satisfied”	
**Determinants of Heterogeneity: Basic Health Worker Characteristics**			
Gender, health worker type, seniority, responsibility, (see Table 1)			Health worker survey
Clinical knowledge	High or intermediate knowledge on pregnancy-related complications (midwives) or common childhood illnesses (nurses, AIS), measured with vignettes^38^			
**Determinants of Heterogeneity: General Work Attitudes**			
Overall work motivation	“In the last 7 days, to what extent were you motivated to work?”	Scale from 0 “not motivated at all” to 10 “completely motivated”	Health worker survey
Autonomous (intrinsic) motivation	Measures with 6 intrinsic motivation and integrated/identified regulation items^37^		
External motivation	Measured with 4 external regulation items pertaining to economic aspects of extrinsic motivation^37^		
**Determinants of Heterogeneity: PBF-Related Factors**			
Perceived supportive supervision	Measured with 4 items, eg, “My supervisor is always there for me when I need help in my work”	Scale from 0 “do not agree at all” to 10 “fully agree”	Health worker survey
PBF training	Having received formal training in PBF	0 = no; 1 = yes	
PBF exposure	Having been working at a PBF facility when PBF was introduced (versus having joined the facility when PBF was already on-going)	0 = no (exposure from later); 1 = yes (exposure from start)	
**Determinants of Heterogeneity: Facility Characteristics**			
Quality of care at intervention start	On 27 structural and process quality dimensions, verified quarterly by the District Health Management Teams through a detailed checklist with over 100 individual indicators^33^; scores theoretically range from 0 to 100	Program data
Quality of care at time of data collection		
PBF equity category	Program facility classification based on staffing levels, remoteness of catchment population, and remoteness from district hospital		1 = most privileged, 9 = least privileged
Number of clinical staff	Total number of clinical facility staff	Facility assessment
Staff-patient ratio	Total number of patients in month before data collection divided by number of clinical skilled staff	

Abbreviations: PBF, performance-based financing; AIS, Agent Itinérant de Santé (preventive services and outreach). ^a^ Only health workers who reported to know the last evaluation results were asked to judge on its fairness ^b^ 27% of the sample (distributed across all cadres, responsibility levels, genders, etc) reported not to receive any bonus payments. However, since the question might have been misunderstood to exclude PBF bonuses, we included in the results shown in Figure 3a only those respondents who reported to receive bonus payments.

####  Analysis

We first performed descriptive analyses of each of the 6 outcome variables. For each, we then employed 3-level (individual, health facility, district) mixed-effects linear (for Likert-type variables as per standard psychometric practice^39^) or logistic (for dichotomous variables) regression to explore determinants of heterogeneity, using the ‘mixed’ and ‘xtmelogit’ commands in Stata 14.2, respectively. Specifically, we modeled associations of the outcome variables with observed individual- and facility-level factors at level 1 as fixed effects, and further accounted for the organizational environment by modeling facility and district random intercepts at levels 2 (health facility) and 3 (district).

###  Qualitative Study Component

####  Design and Sample

 To triangulate and validate the quantitative findings and to better understand observed heterogeneity in PBF knowledge, perceptions, and satisfaction, we performed key informant interviews with the 5 program managers in the MoH PBF unit who had followed program implementation from the start. We opted to interview program managers rather than health workers as in their supervisory role, they were in constant contact with health workers enrolled in PBF and therefore had the best possible oversight over the spectrum of PBF knowledge, perceptions, and satisfaction among the health workforce.

####  Data Collection Process

**Figure 2:**
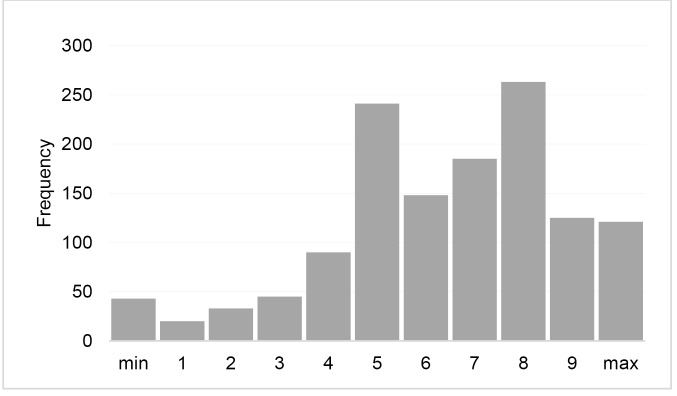
Distribution of Respondents’ Scores Pertaining to Their Overall Satisfaction With PBF. Abbreviation: PBF, performance-based financing.

**Figure 3:**
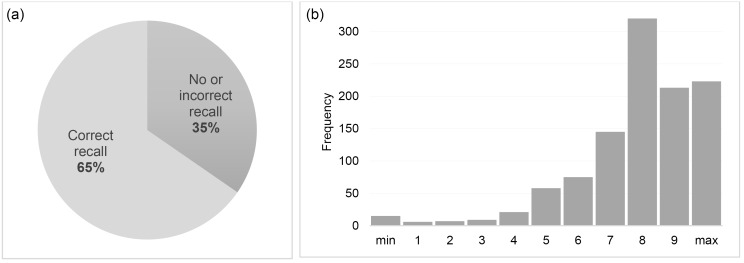
(a) Proportion of respondents having correctly recalled the result of the last performance evaluation result. (b) Distribution of respondents’ perceived fairness scores regarding the performance evaluation process.

**Figure 4:**
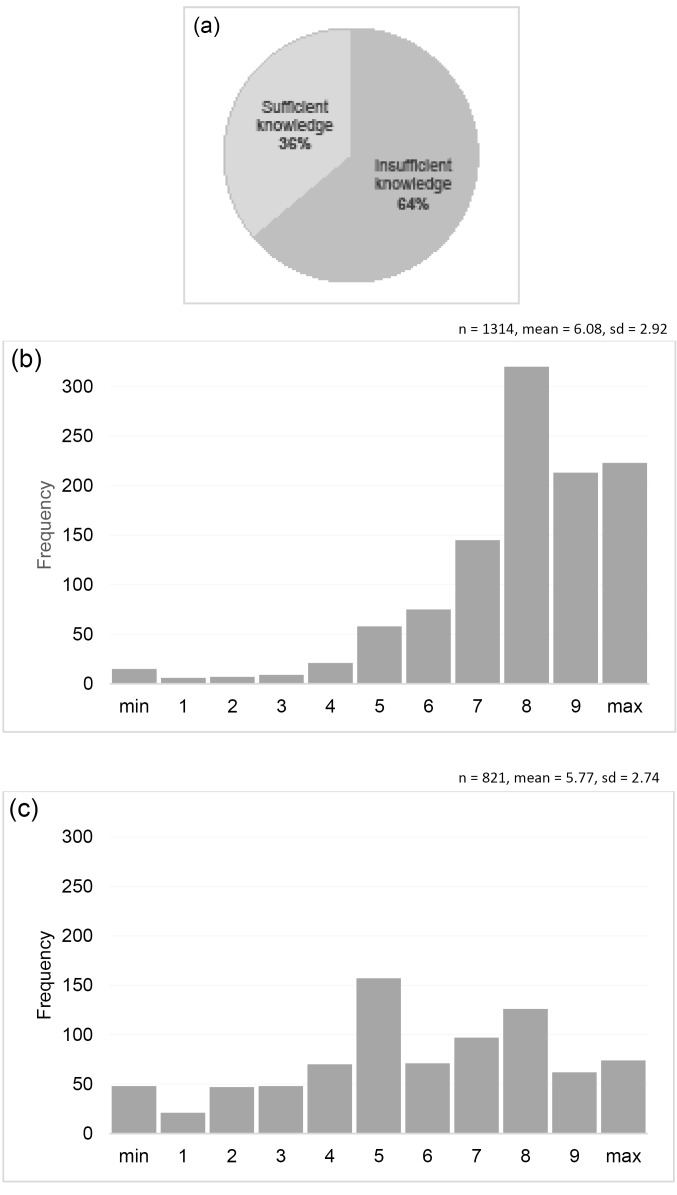
(a) Proportion of respondents having correctly recalled the bonus distribution modalities. (b) Distribution of respondents’ perceived fairness scores regarding the bonus distribution process. (c) Distribution of respondents’ scores pertaining to their satisfaction with their individual bonuses.

The first and the second author conducted all interviews in French, adopting a strategy previously agreed upon by all authors. Respondents were shown the quantitative results presented in Figures 2-4 and asked to comment on them, with interviewers probing for more in-depth information where necessary (“Does this surprise you in any way?”; “Does this correspond to what you have experienced on the ground, or did you have different perceptions?”; “From your perceptions on the ground, what were the reasons for these variations?”). Interviews were audio recorded and verbatim transcribed. Written informed consent was obtained prior to each interview.

####  Analysis

The first and second author independently coded the French material in a mostly deductive process along a predefined codebook, with initial codes that mirrored the quantitative variables in Table 2. The 2 authors further integrated a few new codes that emerged *in vivo* as they proceeded through the transcribed material. The independent analyses advanced by the 2 authors were discussed among all authors and minor discrepancies in emerging interpretations resolved by referring back to the data and/or by relating findings to the context of the intervention. Quotes illustrating main findings were selected and translated from French to English for the purpose of publication.

## Results

 Quantitative and qualitative findings are jointly presented in the following section, organized along 3 main topics: overall satisfaction with PBF; knowledge and perceptions regarding performance evaluation; and knowledge and perceptions regarding individual bonuses.

###  Overall Satisfaction With Performance-Based Financing

Figure 2 shows that health workers were on average moderately satisfied with PBF overall, although with substantial variation. Program managers confirmed that these findings correspond to their own perceptions of health workers’ satisfaction with the intervention.

*“Personally, I think that this [result] is right. It depends on what they experience in each health facility. Some are satisfied because with PBF, they have felt a change. Others are not satisfied because what they expected was not what happened” *(R1).

 Specifically, program managers reported that in their perception, most health workers appreciated PBF for leading to improvements in their work places, for helping them develop their skills, and for improving the care their patients were able to receive. Four out of 5 managers saw these as the most important factors in determining health workers’ satisfaction with PBF. In contrast, one program manager perceived the individual financial incentives as the most important satisfying factor.

*“There are people who are satisfied, who say that regardless of the payment, the positive effect that PBF has on their professional training, on their career, is very beneficial” *(R3).

*“People were able to equip themselves, in terms of medical equipment, construction, there was quite a bit of improvement. That can only increase the level of satisfaction” *(R4).

*“The factor that makes people satisfied is first and foremost the financial motivation. Because today people are too hooked on money” *(R4).

 All respondents underlined that most health workers were also generally happy with the program objectives, indicator set, and procedures.

 At the same time, program managers perceived several factors to have impacted satisfaction negatively, most importantly the following two. First, the substantial delays in payment incurred by the program at various points in time weighed on many health workers’ general satisfaction.

*“There is an aftertaste that has remained from PBF. Many have lamented the late payments and when asking them about their appreciation of PBF, because of that only, they say they are not satisfied” *(R3).

 Second, a number of design changes were made during the course of implementation, most notably a significant reduction in price levels for various indicators and the introduction of a proportional investment requirement, which in combination lowered subsidy amounts both for the facility and particularly for individual staff members. Against this context, one program manager reflected on the importance of starting with realistic price levels.

*“Lessons learned…we need to pay attention to prices. Once they are high and you reduce…unfortunately, we started high, the money ran out, we had to lower prices. It affected [satisfaction]…” *(R2).

 In explaining heterogeneity in overall satisfaction with PBF, program managers underlined the importance of individual differences in general attitudes towards work.

*“The people who are not satisfied are usually those who do not want to work, because when you talk with them, they tell you that with PBF, you write a lot, there is a lot of work to be done, and the money you give us is not much” *(R4).

 They also pointed out that health workers held different ideas and expectations about how the program ought to benefit them, influencing the extent of their overall satisfaction.

*“Those for whom PBF is mostly about money, they will tell you that it is not good because payments are late and so on. However, others for whom it improves and strengthens their skills, allows them to work in good conditions, and so on, they think it’s good and many are in this mindset” *(R3).

Results of the quantitative heterogeneity analysis (Table 3) support this notion, showing that health workers with higher overall satisfaction with PBF tended to have higher general and autonomous (intrinsic) work motivation, but – somewhat contrary to program managers’ perceptions – be generally more motivated by economic considerations.

 Quantitative results further show a positive relationship between perceived supportive supervision and satisfaction with PBF. Health workers in facilities assigned to a higher equity category, signaling disadvantage in terms of geographic remoteness and staffing levels and leading to proportionally higher PBF subsidies and bonuses, also tended to be more satisfied with PBF overall. Beyond this, results show substantial residual variation between districts and health facilities.

**Table 3:** Multivariate Results

	**Overall Satisfaction With PBF**		**Performance Evaluation**		**Bonus Distribution**		**Satisfaction With Earnings From PBF**
			**Knowledge**	**Perceived Fairness**	**Knowledge**	**Perceived Fairness**	
	**Coef.**	***P***	**Coef.**	***P***	**Coef.**	***P***	**Coef.**	***P***	**Coef.**	***P***	**Coef.**	***P***
Health worker characteristics: basic												
Health worker gender: male	-0.10	0.54	**0.75**	**0.00**	0.16	0.27	0.15	0.40	-0.07	0.72	-0.11	0.62
Health worker type (base: nurse)						
Midwife	-0.51	0.81	0.24	0.41	-0.01	0.97	-0.37	0.88	0.01	0.98	0.55	0.07
Assistant midwife	-0.21	0.32	-0.29	0.27	0.12	0.54	**-0.56**	**0.02**	**-1.62**	**0.00**	0.39	0.20
AIS	-0.12	0.92	-0.41	0.10	0.13	0.45	-0.28	0.20	**-0.94**	**0.00**	0.44	0.10
Responsibility: Facility in-charge	0.06	0.76	**0.61**	**0.01**	0.26	0.10	**1.34**	**0.00**	**0.82**	**0.00**	0.33	0.18
Health worker seniority	0.01	0.56	-0.01	0.58	-0.01	0.33	-0.00	0.90	0.01	0.40	0.02	0.28
Clinical knowledge: high/intermediate	-0.21	0.86	0.06	0.78	0.03	0.82	**0.60**	**0.00**	-0.04	0.82	0.18	0.39
Health worker characteristics: general work attitudes												
Overall work motivation	**0.24**	**0.00**	0.04	0.23	**0.10**	**0.00**	0.03	0.28	**0.15**	**0.00**	**0.17**	**0.00**
Autonomous motivation	**0.12**	**0.04**	0.07	0.38	**0.11**	**0.03**	-0.05	0.41	0.01	0.89	-0.10	0.19
External motivation	**0.11**	**0.00**	0.00	0.93	0.02	0.54	-0.04	0.24	**0.12**	**0.00**	**0.13**	**0.00**
Health worker characteristics: PBF-related variables												
Perceived supportive supervision	**0.12**	**0.00**	0.06	0.30	**0.17**	**0.00**	0.07	0.17	**0.28**	**0.00**	**0.17**	**0.01**
PBF training: received	0.01	0.94	0.36	0.07	0.18	0.15	**0.89**	**0.00**	0.27	0.11	0.10	0.58
PBF exposure: from the start	-0.05	0.73	**0.51**	**0.01**	0.22	0.08	-0.06	0.71	0.19	0.24	-0.03	0.89
PBF knowledge: correct/sufficient	-	-	-	-	0.92	0.52	-	-	**0.59**	**0.00**	-0.13	0.48
Fairness perceptions	-	-	-	-	-	-	-	-	-	-	**0.34**	**0.00**
Facility characteristics												
Quality of care at baseline	0.00	0.87	0.02	0.21	-0.01	0.07	0.01	0.50	0.00	0.63	0.00	0.83
Quality of care at data collection	0.00	0.92	0.02	0.15	**0.03**	**0.00**	0.00	0.68	-0.01	0.20	-0.01	0.22
PBF equity category	**0.18**	**0.02**	-0.06	0.62	**0.17**	**0.00**	0.05	0.51	-0.02	0.77	**0.26**	**0.00**
Number of clinical staff	0.00	0.77	**-0.08**	**0.00**	0.01	0.41	-0.01	0.50	-0.02	0.10	-0.01	0.53
Clinical staff-patient ratio	0.00	0.31	-0.00	0.53	0.00	0.64	0.00	0.34	0.00	0.27	0.00	0.57
Cluster-level variance	**Coef.**	**95% CI**	**Coef.**	**95% CI**	**Coef.**	**95% CI**	**Coef.**	**95% CI**	**Coef.**	**95% CI**	**Coef.**	**95% CI**
District	0.30	0.11, 0.80	0.85	0.51,1.41	0.06	0.01, 0.30	0.61	0.38, 1.00	0.17	0.05, 0.58	0.19	0.04, 0.82
Health facility	0.48	0.26, 0.87	1.49	1.19, 1.86	0.44	0.26, 0.75	0.19	0.00, 12.06	0.50	0.25, 0.98	0.82	0.44, 1.54

Abbreviations: PBF, performance-based financing; AIS, Agent Itinérant de Santé (preventive services and outreach).

###  Knowledge and Attitudes Regarding Performance Evaluation 

As Figure 3a shows, two-thirds of health workers were able to correctly recall their facility’s last quality performance score. Program managers were not surprised by this finding.

*“That does not surprise me. Because when we do the evaluations, people are interested because they know they have money in it. So they know [the results]” *(R4).

 Aside from monetary aspects, program managers underlined the competitive element in PBF leading health workers to know their scores.

*“The comparison of quality scores, it touches the ego of the health facility in-charges. When they return to the health facility, they talk about it. And they call each other, we had so much, you, how much did you have? We were better than you! [The scores] remain engraved in the heads of their staff, they know what they had” *(R3).

They further explained that processes are set in a way that all staff members should be informed, even though usually only facility and department management staff participate actively in the verification exercise. In correspondence with this, results of the quantitative heterogeneity analysis (Table 3) show significantly higher knowledge levels among facility in-charges. Quantitative findings further show substantially higher knowledge levels among health workers who had been working in intervention facilities at the start of PBF, and – with marginal statistical significance – who had received PBF training. Findings detected substantial variation by district and particularly by health facility.

 At the same time, program managers voiced disappointment that knowledge levels were not higher. In explaining shortfalls from a 100% knowledge level, they mentioned particularly 3 aspects, beyond individual variation in memory and interest. First, it appeared that not all facilities practiced knowledge sharing as intended, in part because the verification teams did not always spend as much time at the facility as originally intended. Quantitative findings imply that knowledge sharing might be a particular problem in facilities with higher numbers of staff, where knowledge levels were significantly lower. Program managers also pointed at the importance of the facility in-charge’s initiative, ambition, and leadership qualities in this regard. Second, it appears that many health workers were mostly interested in whether their facility surpassed the threshold rendering them eligible for quality bonuses, but did not necessarily recall the exact score. Third, the payment delays might have contributed in that they led to a temporal disconnect between verification results and the amount of bonus to be received, rendering the link less salient and therefore less interesting to health workers.

Figure 3b shows that the majority of health workers perceived fairly high levels of fairness regarding the performance evaluation process. Program managers confirmed this.

*“They think it’s fair, and they find that the evaluators are rigorous and that the things they criticize are justified” *(R2).

Quantitative results indicate no relationship between correct knowledge of evaluation results and perceived fairness (Table 3), but health workers with higher perceived fairness tended to have higher overall motivation, autonomous (intrinsic) motivation, and perceived supportive supervision. Perceived fairness was also higher in facilities with higher actual quality performance level at the time of data collection, and with a higher equity category indicating more severe disadvantage. Controlling for actual knowledge levels, staff who had not been exposed to PBF from the start of the program, when extensive training happened, tended to have lower perceptions of fairness, although this variable only reached marginal significance as a predictor of perceived fairness. One program manager, however, confirmed that complaints had been largely limited to new staff. Similar to what was observed for PBF knowledge, results further indicate substantial variation between facilities and districts.

###  Knowledge and Attitudes Regarding Individual Bonuses

Figure 4a shows that only about one third of health workers had sufficient knowledge about the individual bonus distribution, defined as knowing who had decided on the bonus distribution mode – the PBF program management, correctly answered by 70% – as well as at least 4 out of 5 distribution criteria. Around 80% correctly recalled as distribution criteria salary category, seniority, and days of absence, respectively, whereas level of responsibility (ie, facility in-charge vs. staff member) was only mentioned by 49% and individual performance evaluation by only 34%.

Program managers confirmed this picture, and provided several explanations for the observed gaps in knowledge. Generally, although bonus distribution – using the *outil d’indice* – was intended to be a participatory process, this was not the case in many facilities, with the health facility managers often calculating shares in a non-transparent way. This appears to have somewhat improved over time, but problems persisted throughout the implementation period. Again, program managers underlined the importance of the health facility manager’s personality and leadership competence and style in this regard. Further, they stressed the importance of training in PBF and the general lack thereof for newly affected staff.

*“Is the outil d’indice filled in a participatory way? If health workers were all involved in filling it, they would all know the criteria” *(R2).

Results of the quantitative heterogeneity analysis (Table 3) correspond to program managers’ perceptions in that knowledge levels were substantially higher among health facility managers than regular staff members, and lower for lower-level cadres. Respondents were more likely to have sufficient knowledge when having received training in PBF, and the higher their general clinical knowledge.

 In regards to the criterion of responsibility, program managers explained that since it only pertained to the health facility manager, many regular staff members were not aware of it. In regards to the individual performance evaluation, they reported that lack of awareness resulted from evaluations not being done as prescribed in many facilities. They explained that considering the workload associated with quarterly individual evaluations and the potential for discontent and conflict, many in-charge’s appeared unwilling to comply. In many facilities, it seems that staff had come to an understanding to assign the same performance scores to all staff members. One program manager underlined that not all blame should be put on the health facility managers, however, explaining that higher-level leadership issues – district managers also had not evaluated facility managers as frequently as they should have – and integration into the existing system – where individual performance evaluation is done annual rather than quarterly – also played a role.

Figure 4b shows that despite these knowledge gaps, the majority of health workers indicated fairly high perceived fairness of the bonus distribution mode. Program managers reported a slightly less positive perception of perceived fairness among health workers, but explained that cases of perceived unfairness were mostly due to the issue of transparency introduced above. In support of this, results of the quantitative heterogeneity analysis (Table 3) indicate that perceived fairness was substantially higher among facility managers and among higher-level cadres in general – who were likely more involved and informed –, as well as among health workers perceiving their supervisors to be generally supportive.

 Perceived fairness was also markedly higher among health workers who had sufficient knowledge of the distribution mode and among health workers with higher general and/or external motivation.

Finally, Figure 4c shows large variation in health workers’ overall satisfaction with the individual bonuses they received. Again, program managers again underlined the key role of fairness, transparency, and consensus in application of the criteria, while they perceived absolute amounts earned to be less but not entirely unimportant.

*“All those who do not agree with the bonuses, they find that their in-charges do not distribute transparently, that’s what creates a lot of problems. [...] Those who said they are satisfied are from facilities where they have found a consensus on how to distribute the bonuses. But where there is dissatisfaction, there is no consensus and there is arbitrariness in it so people are not happy. So it depends less on the absolute amount but more on the distribution process” *(R3).

*“There are also people complaining about the amount [...]. This happens in 2 situations. In health facilities with a lot of staff members who share... so what goes to each individual is little. And in very poor performing health facilities that do not receive much” *(R5).

Results of the quantitative heterogeneity analysis (Table 3) confirm the importance of fairness perceptions and positive perceived supervision. Further, health workers with higher overall and external motivation and working in more disadvantaged facilities tended to be more satisfied with the individual bonuses.

## Discussion

Our study makes an important contribution to the literature by being the first to quantify health workers’ knowledge and perceptions of PBF and to systematically explore heterogeneity therein. The results clearly demonstrate that health workers react in very different ways to the same overall intervention. This corresponds to what prior qualitative research in other settings had indicated.^7-19^ Overall satisfaction with PBF was positively shaped by perceived improvements in working conditions induced by PBF, and negatively impacted by the payment delays incurred by the program as well as by various design changes in the implementation period. Overall satisfaction varied with individuals’ general attitudes towards work, their expectations of who would benefit how from the intervention, and perceptions of the health facility managers’ supportiveness and transparency. Satisfaction and perceived fairness of the performance evaluation and bonus distribution process were primarily related to general work motivation as well as perceptions of the health facility manager as supportive and transparent. Knowledge levels tended to be higher among respondents who had received PBF training and/or been exposed to PBF since the start of the intervention, as well as among health facility managers and generally among higher-qualified staff.

 Hereafter, we wish to focus on the 2 main messages to take away from the study, namely on the need for more research on exploring this within-setting heterogeneity demonstrated by the study, and on the importance of supportive, participatory, and transparent management in shaping health workers’ experiences of PBF.

To date, the vast majority of studies on PBF has focused on average intervention effects across all intervention sites. This is particularly true for studies on PBF impact on utilization and quality of health service provision – we know of no study which has explicitly explored variation in impacts within the same setting –, but also for studies focused on processes or intermediate factors such as health worker motivation, with a few notable exceptions.^9,13,16,18,40^ Inspecting impact estimate confidence intervals and reading between the lines of process-focused studies, however, often strongly suggests that this focus on average effects masks substantial within-setting heterogeneity. This is particularly interesting since many impact evaluations have shown no impact of PBF on average, including in Burkina Faso.^34^ Certainly, the effects of PBF on health service provision are a highly complex dynamic in which health workers’ sentiments are only one aspect among many, yet the results of this study support an emerging criticism of current studies on PBF^41^: Instead of investigating average impact in yet another setting, should we not rather focus on heterogeneity within settings and attempt to understand why some facilities or districts are flourishing with PBF, while others make no or negative progress?

In practical terms, the results of this study support some of the best practices which have been propagated by PBF implementation experts for a long time, such as the importance of training health workers properly in principles and practices of PBF, as well as of participation and procedural transparency.^7^ Most importantly, the study underlined the crucial importance of the facility managers’ managerial skills in a change management process as complex as in the case of PBF implementation. This resonates findings from another process evaluation of the PBF intervention in Burkina Faso^40^ and previous findings for instance in Malawi^8^ or Nigeria.^42^ Clearly, in a setting with severe human resources shortages like Burkina Faso, appointing only managers with sufficient managerial skill is not a viable option for sheer lack of qualified candidates to choose from. However, future training measures both within the context of PBF and beyond might want to focus more on training managers not only in technical but also in interpersonal aspects of organizational change processes.

One important limitation of our study is that, as in most cross-sectional psychometric studies,^43^ respondents’ choice of answer is not solely influenced by their underlying satisfaction or fairness perceptions. Rather, answers are also determined by individual differences in interpreting the anchors – at the same underlying satisfaction level, different respondents will likely choose somewhat different numbers of the 0-10 scale –, by social desirability aspects related to the specific interview setting and personality, as well as by other factors such as understanding of the methods. We acknowledge that respondents’ absolute scores are therefore to be interpreted with some care. However, given the large sample where individual differences in answer tendencies are likely to have averaged out as well as the fact that program managers’ perceptions largely corresponded to the quantitative results, we are confident that this has not influenced the overall messages we take away. Further limitations include the cross-sectional nature of the study, which does not allow for true causal inference, and a risk that program managers have had and reported a somewhat skewed picture of health workers’ true feelings about the intervention. Finally, data regarding actual incentive amounts received by individuals were unfortunately of poor quality, so that we were unable to include this certainly relevant and interesting variable in our models.

## Conclusion

 In Burkina Faso, health workers varied greatly in their knowledge of, satisfaction with, and perceptions of PBF 3 years into the implementation. Factors associated with heterogeneity included general work attitudes, management factors, as well as training in and exposure to PBF. Findings imply that investments into staff training on PBF to enhance knowledge and perceived transparency and into manager training on how to support effective organizational change processes might be beneficial to positive staff attitudes towards PBF, which in turn would likely contribute to improving the effectiveness of PBF. Results also underline the value of shifting focus from average intervention effects to within-setting heterogeneity in future research so as to provide policy-makers and program managers hoping to maximize positive impacts of PBF with tangible and constructive information.

## Acknowledgements

 The study used data from the endline survey of the impact evaluation of the PBF program in Burkina Faso. The impact evaluation, including data collection for the health worker survey, was funded by the World Bank through the Health Results Innovation Trust Fund (HRITF). The authors would like to thank all Centre MURAZ staff and interviewers for the extensive fieldwork; the Programme d’Appui au Développement Sanitaire (PADS) for financial support for data collection given to Centre MURAZ; and The World Bank, particularly Paul Jacob Robyn and Ousmane Haidara, for their support with study design, tool development, and data collection, and particularly for allowing us to expand the standard impact evaluation tool kit to include the variables used in this study.

## Ethical issues

 Ethical clearance for the study was obtained from the Ethical Committee of Heidelberg University (S-272/2013) and from the National Ethics Committee in Burkina Faso (N° 2013–7-066 and N° 2015–5-071).

## Competing interests

 All authors were formally engaged in the impact evaluation, which was administered through 2 separate funding streams, one granted to the Heidelberg University Hospital for overall scientific coordination and one for data collection to Centre MURAZ. Specifically, MDA was the Principal Investigator of the IE, but she received no direct payment from the World Bank. JL and JLK were employed at Heidelberg University, working on and funded through the above-mentioned grant, but also received no direct payment from the World Bank (since the grant was managed by the central administration). SS is employed at Centre MURAZ, but received no salary from the World Bank. Analysis and writing presented in this manuscript were conducted beyond the framework of the IE. The World Bank did not interfere with design, data analysis, or writing of this study in any way.

## Authors’ contributions

 JL and MDA conceived the study. All authors contributed to defining the data collection tools and data collection strategy. SMS and JLK supervised the quantitative data collection, with support from JL and MDA. SMS was in charge of data management. JL undertook the quantitative data analysis. JL and JLK conducted the qualitative interviews and analyzed the resulting data. All authors contributed to the interpretation of results. JL wrote the manuscript with contributions from all authors. All authors read and approved the final manuscript.

## Authors’ affiliations

^1^London School of Hygiene & Tropical Medicine, London, UK. ^2^Institute of Global Health, Heidelberg University Hospital and Medical Faculty, Heidelberg, Germany. ^3^Centre MURAZ, Bobo-Dioulasso, Burkina Faso. ^4^UFR/ST, Université Nazi Boni, Bobo-Dioulasso, Burkina Faso.

## Key Messages

**Implications for policy makers**

- In Burkina Faso, health workers varied greatly their knowledge of and satisfaction with performance-based financing (PBF) 3 years into the implementation.
- Knowledge of and satisfaction with PBF varied with general work attitudes, management factors, training in and length of exposure to PBF.
- Findings indicate that investments into staff training on PBF and manager training on organizational change processes will likely be beneficial to positive staff attitudes towards PBF, thereby contributing to improving desired behavior change.

**Implications for public**

The study shows that 3 years into implementations, knowledge of and satisfaction with performance-based financing (PBF) varied greatly among health workers in Burkina Faso. Health workers with more positive general attitudes were found to have higher satisfaction with PBF, but also those who perceived their managers to be more supportive, and those who had either received training in PBF, or had been exposed to PBF from the very beginning of the intervention. The findings imply that investments in systematic training of health workers in PBF and training of managers in managing organizational change processes are likely to result in improved health worker perceptions and satisfaction with the intervention, thereby possibly improving PBF effectiveness.
